# New advances in the treatment of intermediate and advanced hepatocellular carcinoma

**DOI:** 10.3389/fonc.2024.1430991

**Published:** 2024-09-23

**Authors:** Jiang Zhonghao, Yang Fan

**Affiliations:** Department of Hepatobiliary Surgery, The Affiliated Hospital of Inner Mongolia Medical University, Hohhot, China

**Keywords:** intermediate and advanced hepatocellular carcinoma, new progress in treatment, combination therapy, immunotherapy, targeted therapy

## Abstract

Hepatocellular carcinoma (HCC) is the most common primary liver cancer, affecting millions of people worldwide. Due to the complexity and variability of the disease, there are major challenges in the treatment of HCC in its intermediate and advanced stages; despite advances in various treatment modalities, there are still gaps in our understanding of effective therapeutic strategies. Key findings from several studies have shown that the combination of immunotherapy and targeted therapy has a synergistic anti-tumor effect, which can significantly enhance efficacy with a favorable safety profile. In addition, other studies have identified potential biomarkers of therapeutic response, such as tumor protein 53 (TP53) and CTNNB1 (encoding β-conjugated proteins), thus providing personalized treatment options for patients with intermediate and advanced hepatocellular carcinoma. The aim of this article is to review the recent advances in the treatment of intermediate and advanced HCC, especially targeted immune-combination therapy, chimeric antigen receptor T cell therapy (CAR-T cell therapy), and gene therapy for these therapeutic options that fill in the gaps in our knowledge of effective treatment strategies, providing important insights for further research and clinical practice.

## 1 Introduction

Hepatocellular carcinoma is a common malignant tumor, ranking sixth and fifth in incidence globally and in China, respectively. It ranks among the top three in mortality and has long been recognized as a global challenge. HCC is the most common type of liver cancer, accounting for 75% to 80% of cases. Its incidence and mortality vary significantly across regions, which is closely related to risk factors such as viral hepatitis (e.g., hepatitis B and C), alcoholic and non-alcoholic cirrhosis, and fatty liver. Treatment options for HCC include surgical resection, percutaneous anhydrous ethanol injection, Transcatheter arterial embolization (TACE), ablative therapy, chemotherapy and liver transplantation (1). Early stage HCC can be completely cured by surgery, but about 70% of patients have progressed to an intermediate and advanced stage at the time of diagnosis and are unable to undergo surgery. Although liver transplantation is the most effective treatment for liver cancer, many patients fail to meet the Milan criteria and are therefore not eligible for transplantation (2). Therefore, there is an urgent need for more effective treatment options to improve the survival and quality of life of patients with intermediate and advanced HCC. With the deepening of HCC research, a variety of emerging therapeutic approaches, such as targeted immune-combination therapy and combined local and systemic treatment strategies, have begun to be applied clinically.

Common manifestations of intermediate and advanced HCC include tumor enlargement or increased lesions, which increase the complexity of traditional surgical procedures and sometimes make them unfeasible. Localized treatments such as radiofrequency ablation (RFA) and TACE have limited effect at this stage, and while they can mitigate tumor progression, they do not achieve a cure. Treatment of intermediate and advanced HCC has introduced new options, such as targeted agents for lenvatinib and immunotherapy with programmed cell death protein 1 (PD-1)/ligand for PD-1 (PD-L1) inhibitors, but not all patients will benefit; development of drug resistance during treatment may limit therapeutic options and lead to a poor prognosis. In addition, large tumors may further impair liver function, limiting the use of other therapies. HCC is prone to invade intrahepatic blood vessels, especially the portal vein, which increases the risk of cancer cell spread and decreases the likelihood of effective treatment, thereby worsening patient prognosis and making treatment more difficult. In summary, the existing treatment options for HCC are always unsatisfactory, therefore, new advances in the treatment of HCC can provide us with more new ideas on top of the existing multiple treatment options, and help us to solve the problem of HCC more effectively from multiple perspectives and multiple ways. The aim of this article is to analyze and evaluate the therapeutic strategies for intermediate and advanced HCC, including the efficacy, safety, and impact on survival of traditional and emerging approaches such as targeted and immunotherapy and their combinations. By integrating the latest scientific evidence, we seek new strategies to enhance patient survival and quality of life and provide guidance for the treatment of intermediate and advanced HCC.

## 2 Diagnosis and evaluation of hepatocellular carcinoma

The definitive diagnosis of HCC relies on imaging tests such as CT and MRI and tumor markers such as alpha-fetoprotein (AFP), while puncture pathology biopsy is the gold standard for diagnosis. The staging of HCC takes into account the size and number of the tumors, vascular invasion, the state of liver function, and whether or not the patient is experiencing cancer-related symptoms. Although there are various staging systems, such as the Barcelona Clinical Liver Cancer Staging (BCLC) system, the American Association for the Study of Liver Diseases (AASLD) guidelines, and the World Health Organization (WHO) staging system, the BCLC staging system is the most effective way of staging HCC. (3, 4), the BCLC staging system is the most widely used to guide HCC treatment decisions. In the BCLC staging system, early stage HCC refers to stage 0 and A, while intermediate to late stage HCC corresponds to stage B, C and D. Treatment choices need to be adjusted at each stage based on tumor characteristics, patient liver function, and general health. As research progresses, enhanced ultrasound (CEUS), Deoxycholine (DCP), and circulating tumor DNA (ctDNA) assays are also being used in the diagnosis of HCC. ctDNA analyzes DNA fragments released by tumor cells into the bloodstream, providing insight into tumor genetic variation and progression, which is potentially valuable for monitoring response to therapy and recurrence. (5) Treatment strategies and staging systems for HCC may be updated. Therefore, it is crucial to make treatment decisions based on the latest diagnostic criteria, clinical guidelines and research findings, which will help improve the outcome and survival of patients with hepatocellular carcinoma.

## 3 Status and problems of existing treatment programs

### 3.1 Traditional surgical treatment

Hepatectomy is the most common surgical treatment and is indicated when the tumor is confined to the liver and the patient’s remaining liver is functioning well. The goal of the surgery is to completely remove the tumor and a certain amount of surrounding normal liver tissue to ensure that the tumor is completely removed. The resection can range from a lobectomy (removal of a small portion of the liver) to a lobectomy or even a more extensive resection. Patients with intermediate and advanced HCC are often unable to undergo surgical treatment or complete removal of the lesions by hepatectomy because they have progressed to large tumors or multiple lesions that do not meet the indications for hepatectomy, and require adjuvant treatments such as preoperative neoadjuvant chemotherapy or postoperative radiotherapy.

Liver transplantation is another treatment option for patients who cannot undergo liver resection because of the location of the tumor or insufficient liver function. Liver transplantation allows for complete removal of the tumor-containing liver and replacement with a healthy donor liver. However, this approach has specific indications and limitations due to the limited availability of donor livers and the requirement for patients to be on long-term immunosuppression (6). Despite significant advances in imaging techniques, minimally invasive surgery, postoperative management, and liver transplantation, the conventional surgical treatment of HCC still faces the challenges of low early diagnosis rates, high postoperative recurrence rates, shortage of donor livers, and difficulty in individualizing treatment. Future directions for development include improving early screening techniques, exploring new therapeutic approaches, and enhancing individualized treatment to further improve the prognosis and survival quality of HCC patients.

### 3.2 Conventional radiotherapy

The traditional radiotherapy for HCC is external radiotherapy, i.e., CT is used to locate the lesion, and then the radiation generated by radiotherapy equipment irradiates the tumor area from outside the body, thus achieving the purpose of killing cancer cells. However, due to the large size of the liver and rich blood supply, ordinary radiotherapy is more damaging to the liver and other surrounding organs, so it is less applied to HCC patients. Conventional radiotherapy for HCC has made significant progress in technological advances, image guidance, particle therapy and combined therapy, which has improved the precision and effect of treatment. However, challenges such as liver sensitivity to radiotherapy, individual patient differences, high equipment costs, radiotherapy side effects, and long-term efficacy and recurrence are still faced. The future direction of development is to further improve radiotherapy precision, optimize individualized treatment plans, reduce side effects and validate long-term efficacy.

### 3.3 Conventional chemotherapy

Traditional chemotherapeutic drugs such as anthracyclines that can be embedded in DNA base pairs, increasing the distance between base pairs, causing DNA cleavage, interfering with the transcription process and preventing mRNA synthesis. (7) Platinum drugs that can interfere with the replication of DNA in tumor cells, thus killing tumor cells. (8) Fluorouracil, which can inhibit DNA replication competitively to achieve the effect of tumor treatment. (9) The use of platinum-based drugs in the treatment of HCC has been limited primarily because HCC has a low response rate to chemotherapeutic agents and patients may not tolerate chemotherapy-induced side effects. Although chemotherapy continues to have value as an adjuvant treatment option in some cases, the treatment of HCC has expanded with improvements in new strategies and therapies, such as targeted therapy and immunotherapy, which offer more options and hope for patients. Each patient’s condition is unique and therefore requires a customized treatment plan, and future directions for conventional chemotherapy include developing novel chemotherapeutic agents and delivery technologies, optimizing individualized treatment regimens, and exploring effective combinations of chemotherapy with other therapeutic approaches to enhance patient survival and quality of life.

### 3.4 Transcatheter arterial embolization

TACE is a widely used local treatment for patients with intermediate and advanced HCC. It involves the injection of chemotherapeutic drugs into the blood supply arteries of the tumor and the subsequent blockage of these vessels with embolic material, thereby cutting off the blood supply to the tumor and triggering tumor cell death. (10) TACE is not a surgical treatment in the traditional sense, but is an important interventional treatment when dealing with unresectable tumors. Chemotherapeutic drugs and embolic agents are delivered through a catheter directly to the arteries supplying blood to the tumor in order to cut off the tumor’s blood supply and directly kill the tumor cells. TACE is more commonly used in the treatment of intermediate and advanced HCC, but is used in selected early stage patients as a Bridge Therapy to maintain the tumor status until liver transplantation is performed.

### 3.5 Radioactive particle therapy or selective internal radiation therapy

SIRT is a minimally invasive treatment that targets tumors by injecting radioactive microspheres (^90^Y microspheres) through the hepatic artery. SIRT is based on the principle that liver tumors rely on the hepatic artery for their blood supply, whereas normal liver tissue is supplied by the portal vein. Tiny radioactive microspheres injected into the hepatic artery can be transported directly to the tumor tissue and work in the tumor’s microvessels. (11–13) The radiation is delivered directly to the tumor tissue and works in the tumor’s microvessels. This method limits the radiation to the vicinity of the tumor and kills the tumor cells directly, while limiting the radiation exposure to healthy liver tissue to a minimum to minimize damage to normal tissue.

### 3.6 Microwave ablation, radiofrequency ablation, cryoablation, and chemical ablation

MWA and RFA are techniques that destroy tumor cells through localized heating. Microwave ablation (MWA) uses electromagnetic waves, usually at frequencies between 900 MHz and 2.45 GHz, to generate heat. When microwave energy is absorbed by the tissue, it causes water molecules to vibrate rapidly, generating heat, which leads to cell death and tissue necrosis (14). RFA uses radiofrequency energy (usually in the range of 350-500 kHz) delivered through a fine needle electrode into the tissue to generate heat. This thermal energy causes cellular proteins to coagulate and denature, which leads to tumor tissue necrosis (15). Although not strictly surgical, MWA and RFA also play an important role in the treatment of early HCC. These methods ablate tumor tissue by local heating and are indicated for patients with smaller (usually less than 3 cm) and fewer tumors, especially those who are not candidates for surgical resection. In general, MWA may be a better choice for larger tumors or tumors in vascular-rich areas because it can produce a larger ablation area and is less affected by the cooling effect of blood flow. RFA, on the other hand, may be more appropriate for smaller or location-specific tumors, where precise control is needed to minimize damage to surrounding healthy tissue. Cryoablation (freezing and ablation) utilizes a freezing agent, such as liquid nitrogen or argon, which is introduced directly into the tumor site by means of a probe that rapidly cools the tumor tissue to a very low temperature (usually below -20°C) and then thaws it. The rapid cycles of freezing and thawing lead to the formation of ice crystals inside and outside the tumor cells, damaging cell membranes and organelles and thus causing cell necrosis. Chemical ablation usually uses chemicals such as alcohol (ethanol) or acetic acid. These chemicals are injected into the tumor tissue by causing cell dehydration, protein denaturation, and cell necrosis. However, cryoablation and chemical ablation have limited effect on larger or multiple tumors; and may cause damage to normal surrounding tissues during treatment, especially if the tumor is near important blood vessels or bile ducts, leading to local pain, inflammation, and other complications.

### 3.7 Systemic treatment

#### 3.7.1 Targeted therapy

Targeted therapy focus on specific molecules and signaling pathways in tumor cells that play a key role in tumor growth, spread and angiogenesis. Compared to traditional chemotherapy, targeted therapy aims to hit cancer cells more precisely while reducing damage to normal cells. Commonly used drugs in targeted therapy for HCC include Sorafenib and Lenvatinib. Sorafenib, as the first approved multikinase inhibitor, directly inhibits tumor cell proliferation by blocking the cell signaling pathway mediated by RAF/MEK/ERK, and indirectly inhibits tumor cell growth by inhibiting tumor neovascularization through inhibition of vascular endothelial growth factor receptor (VEGFR) and platelet-derived growth factor (PDGF) receptors (16). Lenvatinib is a multi-receptor tyrosine kinase inhibitor, targeting multiple receptors such as VEGFR1-3, fibroblast growth factor receptor (FGFR1-4), PDGFRα, rearranged during transfection (RET), and receptor tyrosine kinase (KIT), which enhances the infiltration of NK cells into tumors and their cytotoxicity, and promotes the expression of cytotoxicity factors in tumor tissues to exert anti-tumor effects. factor expression in tumor tissues and thus exert anti-tumor activity (17) Targeted therapy can be used either alone or in combination. Targeted therapy can be used alone or in combination with other therapies (e.g., surgery, radiotherapy, or chemotherapy) to improve the therapeutic effect. Although targeted therapy has fewer side effects than conventional chemotherapy, it may still cause some adverse reactions, such as rash, high blood pressure, fatigue, and liver function abnormality (18). Therefore, regular monitoring of patient health and tumor response is needed to adjust the treatment regimen in a timely manner.

#### 3.7.2 Immunotherapy

Immunotherapy is a relatively new treatment that aims to use the patient’s own immune system to recognize and attack cancer cells. Unlike traditional treatments such as surgery, chemotherapy and radiation therapy, immunotherapy aims to enhance or restore the immune system’s natural defenses against tumors. Immunotherapy for liver cancer mainly includes immune checkpoint inhibitors and other immunomodulators.

Immune checkpoint inhibitors are the focus of current immunotherapy research in hepatocellular carcinoma, and they generate tumor immune responses by blocking the immune evasion mechanism utilized by tumor cells, disarming the immunosuppressive effect, activating T cell function, and enhancing the immune surveillance and killing ability of T cells against tumors (19) Tumor immune response. Common immune checkpoints include PD-1, PD-L1, and cytotoxic T lymphocyte-associated protein 4 (CTLA-4). Immune checkpoint inhibitors currently approved for the treatment of HCC include PD-1/PD-L1 inhibitors (e.g. sindilizumab and karelizumab, among others) and CTLA-4 inhibitors (e.g., terelizumab) that have been shown to be effective in certain patients with intermediate and advanced HCC (20) that recognize and kill cancer cells by activating the body’s immune system. In addition, for example, CD47, as an important immune escape molecule, binds to SIRPα on macrophages and prevents macrophages from phagocytosing cancer cells; CD24 interacts with Siglec-10 on macrophages and provides inhibitory signals to prevent phagocytosis; and the ability of the immune system to remove tumor cells can be significantly enhanced by blocking the CD47-SIRPα/CD24-Siglec-10 pathway. immune system’s ability to clear tumor cells (21) which is a key target in cancer therapy.

The choice of immunotherapy is usually based on the patient’s specific circumstances, including the characteristics of the tumor, the patient’s overall health, and prior treatments received. Immunotherapy can be used as a first- or second-line treatment option or in combination with other treatments, such as targeted therapy.

Side effects of immunotherapy may differ from those of conventional treatment, including immune-related side effects such as rash, enteritis, hepatitis, and endocrine abnormalities (22). Therefore, the patient’s health needs to be closely monitored during treatment to detect and manage these potential side effects in a timely manner.

### 3.8 Supportive therapy

Supportive care refers to a range of therapeutic measures aimed at improving patients’ quality of life, relieving symptoms and reducing side effects associated with cancer treatment. This treatment does not directly target the tumor itself, but rather focuses on the overall well-being of the patient, including pain management, nutritional support, psychosocial support, and management of treatment-related complications (23). For patients with intermediate and advanced liver cancer, supportive care also includes the provision of end-stage care to ensure that patients are cared for with dignity and comfort in the final stages of life. End-stage care focuses on symptom control, psychological and emotional support, and the provision of necessary information and resources for patients and families.

The treatment of intermediate and advanced HCC faces multiple challenges, including extensive tumor spread and limited liver function in patients. Although TACE and systemic therapies such as lenvatinib provide some survival benefit to standard treatment, problems such as limited efficacy and side effects remain. Treatment strategies are becoming increasingly personalized with a better understanding of disease mechanisms and the development of new therapeutic approaches. A combination of different therapeutic approaches can provide the best possible outcome for patients, while emerging treatment strategies and clinical trials offer hope for improving patient prognosis. In the field of intermediate and advanced HCC treatment, significant research advances have been made in recent years, and future therapeutic directions are unfolding.

## 4 Research progress on the latest treatments

### 4.1 New advances in targeted therapy

Discovery of new targets: with the deeper understanding of the molecular mechanisms of HCC, the discovery of new targets has facilitated the development of second-generation targeted drugs. These include ramucirumab, an antibody to VEGFR-2; cabozantinib and regorafenib, inhibitors of multiple tyrosine kinases such as mesenchymal-epithelial transition factor (MET), VEGFR2 and RET (24) The RESORCE trial showed (25) that regorafenib significantly prolonged overall survival in HCC patients who failed sorafenib therapy. Niraparib: Niraparib is a polyadenosine diphosphate ribose polymerase (PARP) inhibitor that targets the DNA repair pathway (26), has demonstrated potential therapeutic efficacy in a subset of HCC patients and is currently undergoing extensive clinical studies.

In addition new therapeutic targets are being discovered, for example, new targeted drugs against signaling pathways such as fibroblast growth factor 19(FGF19), MET and VEGF (27) are being developed and tested. These drugs aim to overcome the limitations of earlier drugs, such as improving efficacy, reducing side effects, and addressing drug resistance; however, their research is still ongoing and their efficacy and safety need to be confirmed in additional clinical trials.

### 4.2 Expansion of immunotherapy

Combination Immunotherapy: The combination of PD-1/PD-L1 and CTLA-4 inhibitors has demonstrated superior efficacy enhancement in a variety of cancer treatments. Specifically, the combination of Nivolumab and Ipilimumab combines the mechanisms of action of both PD-1 and CTLA-4 immune checkpoint inhibitors. This strategy aims to significantly enhance the immune response against tumors by simultaneously disarming two inhibitory sites on T cells. This innovative combination is based on the results of the Phase I/II CheckMate-040 Cohort 4 trial. (28) This innovative combination regimen is based on the results of the Phase I/II CheckMate-040 cohort 4 trial, which demonstrated significant efficacy in a subset of HCC patients and has been approved in several regions for the treatment of patients with intermediate and advanced HCC who have received prior therapy (29).

New immune checkpoint inhibitors: including Relatlimab, which targets the lymphocyte activation gene 3 (LAG-3), which significantly enhances immune responses to tumors by activating T cells through inhibition of LAG-3 (30) A phase II trial of PD-1 in combination with LAG-3 is underway (https://clinicaltrials.gov/ct2/show/NCT04567615). T-cell immunoreceptor photoprotein (TIGIT), a key immune checkpoint, is involved in regulating immune response. By inhibiting TIGIT, the ability of the immune system to fight against tumors can be strengthened, and inhibitors related to this are currently under development (31). Meanwhile, studies on other immune checkpoints such as T-cell immunoglobulin domain and mucin domain 3 (TIM-3) and V-domain Ig suppressor of T cell activation (VISTA) are also underway (32) that these molecules play a crucial role in regulating T cell activity and promoting a broad immune response.

Regulation of the tumor microenvironment (TME): the tumor microenvironment includes immune cells, mesenchymal stromal cells, extracellular matrix (ECM), angiogenesis, and various signaling molecules that provide important support for tumor growth, metastasis, and immune evasion. There are current studies regulating tumor-associated macrophages (TAMs): by targeting TAMs with specific molecules or antibodies, it is possible to shift them from a tumor-promoting phenotype to an anti-tumor phenotype, enhancing the immune system’s ability to clear tumors. Unstiffening the ECM: stiffening and reorganization of the ECM promotes tumor proliferation and metastasis. The use of drugs that alter the composition of the ECM or degrade ECM components can reduce the invasiveness and metastatic ability of tumor cells. Targeting cancer-associated fibroblasts (CAFs) to inhibit the activity of CAFs: CAFs promote tumor growth and immune evasion by secreting growth factors and cytokines. Targeting CAFs reduces their tumor-supporting effects (33).

### 4.3 Research on combination therapies

#### 4.3.1 Targeted immune-combination therapy

Research by Richard S Finn et al. has shown (34) that targeted drugs can alter the TME to make tumors more readily recognized and attacked by the immune system, making the use of targeted therapy in combination with immunotherapy a promising direction. For example, the combination of Atezolizumab and Bevacizumab: Bevacizumab targets VEGF, which blocks tumor angiogenesis, while atezolizumab enhances the immune system’s attack on cancer cells by blocking PD-L1. This combination therapy has been shown to have significant efficacy in untreated patients with intermediate and advanced HCC in multiple clinical trials (35) and has been approved as a first-line treatment option for such patients.

**Figure 1:**
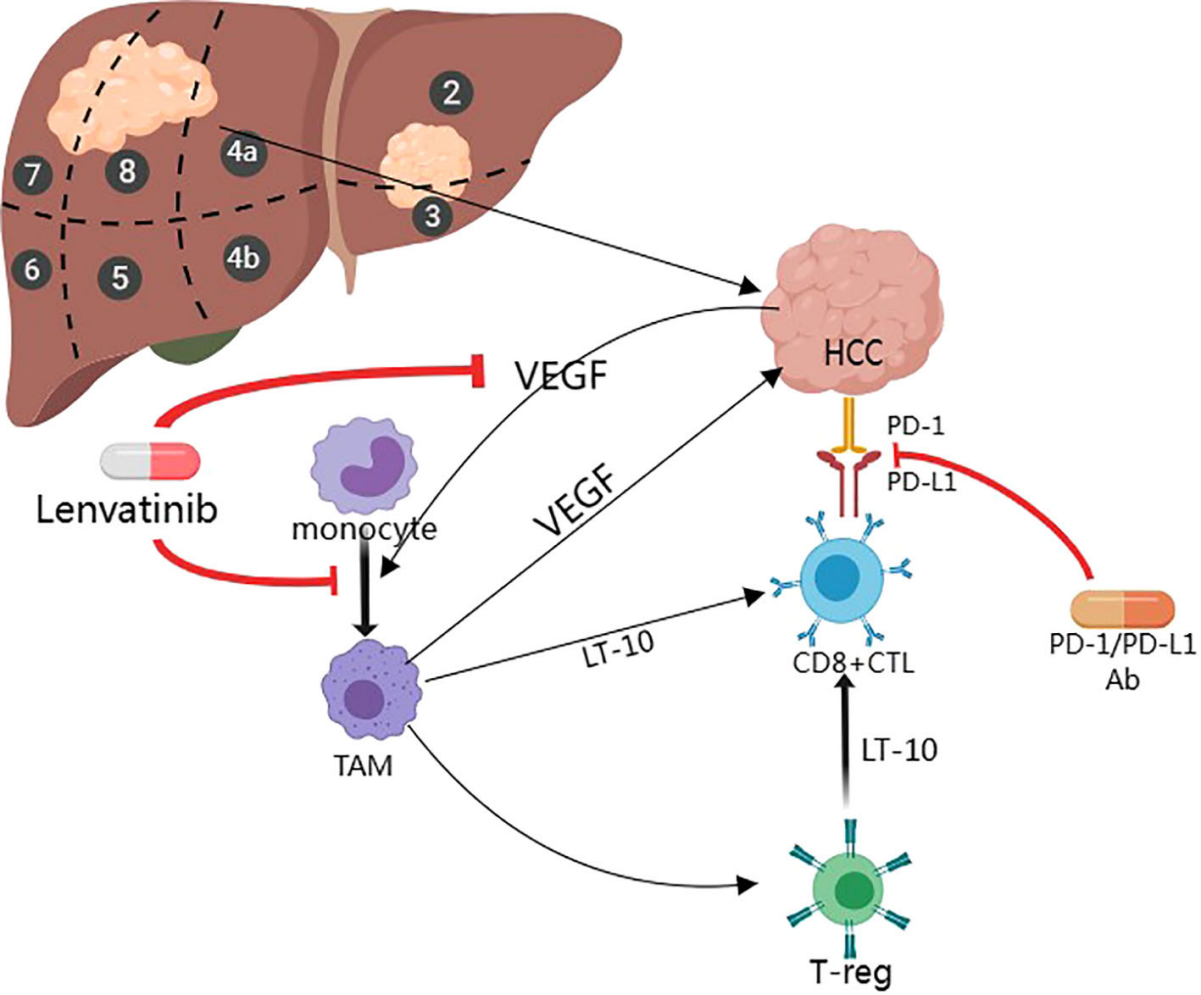
Mechanisms of Lenvatinib in combination with PD-1 Inhibitors for the treatment of HCC (Created with MedPeer).

Combination of Lenvatinib in combination with Carrelizumab and Lenvatinib in combination with Sintilimab: the angiogenesis inhibitory effect of Lenvatinib combined with the immune-boosting effect of Carrelizumab/Sintilimab may improve the microenvironment. As shown in **Figure 1**, Lenvatinib can improve the tumor microenvironment and make it more conducive to immune system attack by targeting angiogenesis and enhancing the anti-tumor activity of T cells. In addition, there is an increase in response rate: combination therapies may improve tumor response rate to treatment and prolong progression-free survival (PFS) and overall survival (OS). This combination therapy could theoretically improve the outcome of HCC (36), and its efficacy and safety are being evaluated through clinical trials.

#### 4.3.2 Combination of local and systemic therapy

Hepatic arterial infusion chemotherapy (HAIC) combined with lenvatinib: significant progress in the study of hepatic arterial infusion chemotherapy combined with targeted therapy and immunotherapy in recent years. Yu Haidong et al. (37) demonstrated that hepatic arterial infusion chemotherapy combined with lenvatinib showed good safety and preliminary efficacy in the treatment of stage B or C HCC.

TACE and its combination regimens: a study has (38) confirmed that TACE combined with HAIC has better therapeutic benefit in patients with HCC with portal vein cancer thrombosis and in patients with giant unresectable HCC. In 2022, a multicenter real-world study conducted by Hong Korea’s team (39) A multicenter real-world study conducted by Hong’s team in Korea in 2022 confirmed that lenvatinib in combination with DEB-TACE was well tolerated and safe, and had advantages over lenvatinib monotherapy in improving OS, PFS, and ORR in patients with intermediate- and advanced HCC. The study and meta-analysis by Hu Zexin et al. (40) Hu Zexin et al.’s study and meta-analysis also confirmed that TACE combined with MWA was superior to TACE alone in improving OS and had a better prognosis for patients with HCC >5 cm. 2023, CHANCE001 study published the results of TACE combined with lenvatinib (41, 42) published results that the triple combination regimen of TACE in combination with lenvatinib and karelizumab showed potential benefits in the treatment of intermediate and advanced HCC, providing a possible combination therapy strategy.

## 5 Future prospects and challenges

Intermediate and advance HCC Research in the treatment area is advancing rapidly with promising future directions involving new therapeutic approaches, more precise molecularly-targeted therapies and immunotherapeutic strategies, and personalization of treatment. Below are some of the key research advances and possible future directions for treatment:

### 5.1 Precision medicine

#### 5.1.1 Personalized therapy based on molecular signatures

By analyzing key genes and proteins in HCC, such as TP53 (which is closely associated with hepatocellular carcinoma development), CTNNB1 (which is key to the Wnt/β-catenin signaling pathway, and whose mutation promotes tumor growth), and TERT the catalytic subunit of telomerase reverse transcriptase (TERT, whose mutation in the promoter region promotes cellular immortalization), and heat Shock Protein (HSP70, high expression levels correlate with tumor aggressiveness and poor prognosis), allowing individualized treatment plans to be tailored for each patient (43).

#### 5.1.2 Application of biomarkers

In addition to conventional indicators such as AFP, new biomarkers are being developed and validated to more accurately predict treatment response and monitor disease progression. For example, the ALBI score is a novel scoring method for evaluating liver function (44), which eliminates the influence of some subjective factors and quantifies the extent of liver damage by bilirubin and albumin levels. It is calculated as (log10 bilirubin) × 0.66 + (-0.085 × albumin); ALBI grade 1: ≤ -2.60, minor liver damage; ALBI grade 2: -2.60 < ALBI ≤ -1.39, moderate liver damage; ALBI grade 3: ALBI > -1.39, severe liver damage.

### 5.2 Exploration of new treatments

#### 5.2.1 CAR-T cell therapy

CAR-T therapy represents a revolutionary advance in cancer treatment (45). The therapy works by extracting a patient’s T-cells, genetically engineering them in the laboratory to give them the ability to recognize and destroy cancer cells, and then injecting these modified T-cells back into the patient. CAR-T therapy is primarily used to treat specific blood cancers, and although its use in solid tumors is still in the exploratory phase, research is actively underway for HCC. For HCC, early stage clinical trials are exploring new targets (46) such as glycoprotein 3 (GPC3) - a molecule that is highly expressed on most HCC cells, as a potential therapeutic target (47) GPC3 is the first molecule that is highly expressed on most HCC cells and is a potential therapeutic target. (48) Constructed GPC3-targeted CAR-T cells for the first time and demonstrated that GPC3-targeted CAR-T cells can effectively inhibit the growth of HCC cells both *in vitro* and *in vivo*, which may open new pathways for treatment This may open a new pathway for treatment.

#### 5.2.2 Tumor vaccines

By activating the immune system, especially T cells, tumor vaccines are able to recognize and attack cancer cells while minimizing damage to healthy cells. Current tumor vaccines fall into the following main categories: tumor cell-based vaccines, which use the patient’s own or another person’s tumor cells that have been specially treated to enhance the immune response. Peptide-based vaccines, on the other hand, use specific protein fragments (peptides) selected from tumor-associated antigens to trigger an immune response to those antigens. DNA and RNA vaccines are designed to stimulate the immune system’s response to specific tumor antigens by injecting DNA or RNA containing the genes that code for those antigens. Viral vector vaccines use modified viruses to carry tumor antigen genes into the body and activate an immune response against the tumor. For HCC, research is underway to develop vaccines that target specific antigens to activate the patient’s immune system against the tumor.

#### 5.2.3 RNA interference, CRISPR/Cas9 and circRNAs

RNA interference is a technique that utilizes small molecule RNA (e.g., small interfering RNA, siRNA) to silence the expression of specific genes. By designing siRNAs with specific sequences, the expression of specific genes in tumor cells can be specifically reduced or inhibited, thereby blocking molecular pathways associated with HCC tumor growth, invasion, or drug resistance (49).

CRISPR/Cas9 is a gene editing technology that enables precise cleavage and modification in the cellular genome. CRISPR/Cas9 technology shows a wide range of prospects for application in the field of HCC research and therapy: 1. Knocking out or adding specific genes to investigate their role in HCC progression; 2. Repairing oncogenic mutations in HCC cancer cells or enhancing the ability of immune cells to form new therapeutic strategies; 3. as a gene therapy tool to directly correct diseased genes in patients, providing possible cures (50). Using these gene editing techniques to target tumor promoters or repair tumor suppressor genes provides new strategies for HCC treatment.

The study of circRNAs in HCC shows great potential to influence the occurrence and development of HCC by regulating gene expression, interaction with proteins, and translation into small peptides. circRNAs’ high stability makes them potential markers for the diagnosis and prognosis of HCC. Currently, there are studies utilizing RNA interference technology to target degradation of oncogenic circRNAs and to develop circRNA-based drug delivery systems. (51) However, the challenges of specificity, safety and efficient delivery of circRNAs need to be further studied and solved. With technological advances, circRNAs are expected to become an important tool in the treatment of HCC.

#### 5.2.4 Nanotechnology

The surface of nanoparticles can be chemically modified by attaching various ligands (e.g. antibodies, peptides or small molecules) to enhance their targeting properties so that they can recognize and bind to specific cancer cells, and thus can be widely used as drug carriers to deliver chemotherapeutic drugs or targeted drugs directly to the tumor region, e.g. hydrogels, macrogels etc. Such targeted delivery can significantly reduce the damage of drugs to healthy tissues, enhance the targeting effect of drugs and reduce their toxic side effects. In addition, magnetic nanoparticles can generate heat under the action of an external magnetic field, and this technology has been applied to magnetothermal therapy. (52) This technology has been applied to magnetothermal therapy. The heat generated in this way selectively destroys tumor cells while minimizing damage to surrounding normal tissue.

#### 5.2.5 Artificial intelligence

AI can use deep learning algorithms to analyze imaging data (e.g., CT, MRI, and ultrasound) to help physicians more accurately identify and locate lesions, improving diagnostic accuracy and efficiency. AI can also analyze clinical data, pathology images, and genomic data to predict the survival, recurrence risk, and therapeutic response of HCC patients to inform personalized treatment. In addition, AI can monitor HCC patients’ disease changes using patients’ clinical data and biomarkers to detect recurrence and metastasis in a timely manner and guide subsequent treatment and management utilization.

#### 5.2.6 Multidisciplinary team

MDT emphasizes collaboration between experts in the fields of oncology, hepatology, radiology, pathology, and basic sciences for patients with HCC to consider and analyze the condition from multiple perspectives and to understand and treat HCC in an integrated manner.

## 6 Conclusion

The treatment of intermediate and advanced HCC is a complex and multifaceted field that requires continuous research and innovation to improve patient survival and quality of life. Current research advances have shown that combination therapeutic strategies, especially targeted immune-combination therapy, offer new hope for patients with intermediate and advanced HCC. Future research needs to further explore the optimal combination, dosage and treatment plan of these therapies, as well as patient-individualized treatment plans to achieve the best possible treatment outcome. In addition, for new therapies for intermediate and advanced HCC, although CAR-T therapy, circRNAs therapy and nanotechnology have shown good results for tumors, there are off-target effects, non-specific responses and safety issues that need to be further addressed to improve the overall effectiveness of HCC treatment.
